# Supplementary material for: Design and evaluation of a simulated wound management course for postgraduate year one surgery residents
Source: PeerJ. 2021 Apr 14;9:e11104. doi: 10.7717/peerj.11104 (PMC8052975; doi:10.7717/peerj.11104)
Supplement: Supplemental Information 2 [file peerj-09-11104-s002.docx]

**Wound management course**

**Participants:** PGY1s

**Duration:** 240 minutes

**Goal:** PGY1s can be able to conduct debridement and ultimately close the wound.

**Course design:**

**Component 1. Baseline** **Test (10** **Minutes)**

Contents: Ask PGY1s to perform a complete excision of the "dumbbell" necrotic tissue and close the wound.

Objectives of this session:

Observe the PGY1s’ performances to have a deeper understanding of their knowledge, skills and attitude about wound management, calibrate the learning direction and teaching strategies if it is necessary.

**Component 2. Interactive Learning (60 Minutes)**

Contents: Encourage PGY1s to review and interpret their performance on the blackboard and share their thinking process and selection basis.

Objectives of this session:

1. Provide immediate, specific and constructive feedback for baseline test
2. Concepts of debridement and closure
3. Motivate their perception, interpretation and construction of wound management as a facilitator, encourage them to communicate and collaborate with each other.

**Component 3.** **Basic Skills Practice (80 Minutes)**

Contents: Complete the closure of round, square, and triangular wounds.

Objectives of this session:

1. Offer hands-on deliberate practice focusing on practical issues
2. Correct their basic skills (behaviors), discern possible problems, observe and assess their understanding of Component 2, encourage them to communicate and collaborate with each other.

**Component 4.** **Reflective Learning (90 Minutes)**

Contents: Continued to complete round, square, and triangular wound closures, or more complex and challenging wounds.

Objectives of this session:

1. Provide feedback based on the completion of Component 3, expand the wound closure content, focusing on the principles and concepts of various local flaps.
2. Encourage them to communicate and collaborate with each other, and offer help to others as the instructor.
