# Supplementary material for: Design and evaluation of a simulated wound management course for postgraduate year one surgery residents
Source: PeerJ. 2021 Apr 14;9:e11104. doi: 10.7717/peerj.11104 (PMC8052975; doi:10.7717/peerj.11104)
Supplement: Supplemental Information 3 [file peerj-09-11104-s003.docx]

Results of process measure

| Dimensions | Mean+SD | | |  | *p*-value | | |
| --- | --- | --- | --- | --- | --- | --- | --- |
|  | 2014 | 2015 | 2016 |  | 2014 vs  2015 | 2014 vs  2016 | 2015 vs  2016 |
| Maintaining a sterile field | 7.56+0.79 | 7.82+0.89 | 7.82+0.21 |  | 0.199 | 0.193 | 0.977 |
| Knowledge and handling of instrument | 7.49+0.92 | 8.11+0.47 | 7.78+0.52 |  | 0.001 | 0.113 | 0.060 |
| Quality of excision | 7.58+0.78 | 7.62+1.58 | 7.84+0.56 |  | 0.894 | 0.392 | 0.441 |
| Quality of debridement | 7.23+0.95 | 7.68+0.85 | 7.84+0.58 |  | 0.049 | 0.008 | 0.433 |
| Dissociation of subcutaneous tissue | 0 | 6.23+2.35 | 7.56+0.79 |  | 0.000 | 0.000 | 0.001 |
| Quality of suturing and knots | 7.14+0.91 | 7.60+0.64 | 7.56+0.82 |  | 0.043 | 0.065 | 0.855 |

Note：p values were calculated by the least significant difference tests
